# An RSS Transform—Based WKNN for Indoor Positioning

**DOI:** 10.3390/s21175685

**Published:** 2021-08-24

**Authors:** Rong Zhou, Yexi Yang, Puchun Chen

**Affiliations:** School of Control and Computer Engineering, North China Electric Power University, Beijing 102206, China; 120192227084@ncepu.edu.cn (Y.Y.); 120202227078@ncepu.edu.cn (P.C.)

**Keywords:** Wi-Fi fingerprint, RSS fluctuation, AP selection, WKNN

## Abstract

An RSS transform–based weighted k-nearest neighbor (WKNN) indoor positioning algorithm, Q-WKNN, is proposed to improve the positioning accuracy and real-time performance of Wi-Fi fingerprint–based indoor positioning. To smooth the RSS fluctuation difference caused by acquisition equipment, time, and environment changes, base Q is introduced in Q-WKNN to transform RSS to Q-based RSS, based on the relationship between the received signal strength (RSS) and physical distance. Analysis of the effective range of base Q indicates that Q-WKNN is more suitable for regions with noticeable environmental changes and fixed access points (APs). To reduce the positioning time, APs are selected to form a Q-WKNN similarity matrix. Adaptive K is applied to estimate the test point (TP) position. Commonly used indoor positioning algorithms are compared to Q-WKNN on Zenodo and underground parking databases. Results show that Q-WKNN has better positioning accuracy and real-time performance than WKNN, modified-WKNN (M-WKNN), Gaussian kernel (GK), and least squares-support vector machine (LS-SVM) algorithms.

## 1. Introduction

Indoor positioning is used in areas where the global positioning system (GPS) is not desirable. Unlike the well-solved outdoor positioning problem, indoor positioning encounters the challenge of no line of sight (NLOS). However, the extensive deployment of wireless infrastructure and the proliferation of mobile devices have facilitated positioning in indoor scenes, and positioning based on received signal strength (RSS) has been an attractive solution [1]. Common wireless signals such as Bluetooth [2], Wi-Fi [3], ultra-wideband (UWB) [4], and radio frequency identification (RFID) [5] are often used for positioning. Positioning is also dependent on the existing position calculation algorithms, such as direct positioning, geometrical calculations, and fingerprint localization. In terms of measurement techniques, the common methods include time of arrival (TOA), angle of arrival (AOA), time difference of arrival (TDOA), and received signal strength (RSS) [6]. Among these methods, Wi-Fi—based indoor positioning has gone viral for advantages such as no need for additional hardware assistance except access points (APs) [7], adaption to various indoor environments, and convenient acquisition of RSS.

Building an RSS fingerprint database for comparison with test points (TPs) is the core of RSS fingerprint-based indoor positioning. Different points in the positioning area receive different RSSs of each AP, which compose fingerprints, and these different detailed characteristics provide unique confirmation information. Positioning usually has offline and online phases. The main work in the offline stage is building a fingerprint dataset based on the measured RSS of APs at different spots (i.e., reference points, or RPs), which are predefined according to the AP’s position, the expected positioning accuracy, and the area of the whole positioning field. It is time- and labor-consuming. To address this issue, researchers have done some significant work: (1) the large UJIIndoorLoc dataset covers multiple buildings [8]; (2) the IPIN2016 Tutorial dataset focuses on small scene positioning [9]; and (3) Zenodo dataset contains both long- and short-term changes [10]. The position coordinates are estimated in the online stage by matching the newly minted fingerprint of a TP with multiple known fingerprints of reference points stored in a well-built database.

Calculating the similarity between a dataset’s fingerprints and a TP’s fingerprint to estimate its position is the basic idea of matching. Positioning accuracy is affected to various degrees by the quality of the estimation algorithm and the reliability of the fingerprint dataset.

Among many indoor position estimation algorithms, those employing machine learning are the subject of much research. The K-nearest neighbors (KNN) algorithm, a popular machine learning method, was first introduced in positioning, and algorithms including WKNN, M-WKNN, and GK are based on it [11,12,13,14]. Other machine learning methods applied for indoor positioning include support vector machine (SVM) [15,16], k-means clustering [17], and deep neural networks [18,19,20].

RSS fluctuation is one of the important reasons that reduce the reliability of fingerprint dataset. In fact, there are many factors that cause RSS fluctuation. Signals would fade in propagation, mainly including fast fading and slow fading, which are different but not independent. Slow fading is mainly due to path loss and is related to moving speed and working frequency of electromagnetic wave. The phenomenon that the multipath transmission of the signal causes the rapid fluctuation of the received signal is called fast fading [21]. Furthermore, individual phone models and individual electrical devices like iPad have different sensitivities, and the RSS indicated on one may vary. Meanwhile, APs adjust their power level according to the environment and traffic load. Multiple input multiple output (MIMO) technology adopted in modern APs also drastically alters the radio frequency field in a time-varying manner. Affected by all these factors, a completely reliable fingerprint database has not been built yet. Some studies treat signal fluctuation as noise, with promising results [22,23,24,25,26,27,28,29]. However, unified processing as noise would erase some characteristics of the signal, which is not conducive to the improvement of positioning accuracy. Therefore, we correct signal fading according to the signal propagation law and smoothen the RSS fluctuation to preserve the signal characteristics as much as possible. This smoothing method has a higher tolerance to different path loss factors, does not require frequent recalibration, and retains the difference information of RSS.

Many APs are easily detected in indoor public places, but some APs with empty or low values (named useless APs) affect the reliability of the fingerprint database. In an ideal environment, more APs would mean higher positioning accuracy. However, in actual positioning, it means more signal loss and more fluctuations. In addition, superabundant APs would consume more computing resources [30]. Therefore, mass APs must be selected reasonably. The selection criterion is to keep as many effective features as possible while removing the AP’s RSS, which is useless for improving the positioning accuracy. We combine the maximum value of RSS with the occurrence frequency of effective signals and propose a new standard for AP selection, which ensures accuracy and improves the efficiency of the algorithm.

Our work has four parts:

- After analyzing the relationship between RSS and physical distance in signal propagation, base Q is proposed to smooth fluctuation by transforming RSS before the similarity match;
- A new AP selection method is proposed, which selects APs that contribute more to the positioning;
- An adaptive K value is proposed, which is dynamically determined according to the distance collection S between RPs and TP;
- Based on the above three parts of this work, the Q-WKNN algorithm is proposed. The algorithm is compared to commonly used algorithms such as WKNN, M-WKNN, GK, and LS-SVM to demonstrate its improved positioning accuracy and real-time performance. The environment where the Q-WKNN algorithm could achieve better position results is found.

The rest of this article is structured as follows. Related work is reviewed in Section 2. A detailed description of the proposed algorithm Q-WKNN is given in Section 3. Experimental methods and settings are discussed in Section 4, and the proposed algorithm is compared with several well-used algorithms in positioning accuracy and time-consumption. Section 5 summarizes our work and concludes that the Q-WKNN algorithm can effectively improve the positioning accuracy.

## 2. Related Work

### 2.1. Processing for RSS Fluctuation

RSS fluctuations are often treated as noises. Taking the mean of successive measurements at the same RP was historically utilized to deal with signal fluctuations [22]. In this case, the signal fluctuation is treated as simple additive noise, and the fluctuation is simply eliminated by the operation of averaging. It is effective, but the results are not desirable. Some studies have examined signal fluctuations in detail to extract more useful information from data affected by multipath effects, environmental dynamics, and equipment difference.

The mainstream ideas put forward to deal with signal fluctuation consist of fingerprint structure reconstruction and signal transformation before analysis. Reconstructing the fingerprint structure is often based on the relationship between fingerprints. An NR-RSS fingerprint based on the RSS difference between adjacent positions was constructed to eliminate the influence of environmental dynamics and equipment heterogeneity [23]. Using the spatial relationship between fingerprints in multiple adjacent positions, a fingerprint spatial gradient (FSG) was proposed to reduce the uncertainty of RSS fingerprints [24]. These fingerprint structures’ reconstructions are often subject to complex calculations. There are several ways to transform the RSS. For example, time-domain convolution was applied to model the dynamic multipath behavior, making it linearly separable, and extracting the robust signal characteristics [25,26]. This achieved terrific results, but fluctuation that can reflect the characteristics of RSS is also considered as multipath interference. Another promising way to deal with fluctuation is choosing the appropriate data transformation rather than using RSS values directly to smooth the fluctuation. Because of the logarithmic property of signal propagation, the linear transformation, such as normalization, can be outperformed by exponential transformation [27,28]. Accordingly, lowest RMSE could be achieved after exponential transformation, but a base that considers path loss parameters and signal fluctuations is difficult to determine [29].

We combine the ideas of separation and simplification. First, we eliminate the coarse noise caused by environmental mutation. Then, inspired by mean smoothing [22] and exponential transformation [26], we smooth the RSS fluctuations after a Q-based RSS transformation on the premise of retaining RSS characteristics according to the signal propagation model.

### 2.2. AP Selection

A reasonable choice of APs improves both the efficiency of the algorithm and the positioning accuracy. Particle swarm optimization was used to generate AP placement strategies for different maps [30]. Feature selection was applied to intelligently select the number of AP for location estimation using fewer APs [31]. Discriminability APs were measured independently, ignoring correlation between APs. An intelligent selection method combining AP position information was subsequently proposed [32]. The idea of information gain has achieved outstanding results, but the computational cost is high. A selection method considering the RSS standard deviation (SD) of APs in the online positioning stage was proposed to filter out some abnormal APs of RSS [33]. In fact, AP prescreening in the offline stage should not be ignored. AP discrimination indices and AP strength were used to perform AP selection in offline and online phases, respectively, with better results than the commonly used Fisher and maximum RSS strength selection [34]. However, AP discrimination indices may be difficult to distinguish with a large amount of randomly distributed APs.

Choosing the most robust AP is inseparable from signal strength characteristics. It has been proposed that taking the average RSS value as the signal strength feature is not the best choice [35]. Our AP selection strategy combines the maximum RSS, which reflects the individual strength of APs, with the appearance ratio of effective signals to choose the reliable APs.

### 2.3. Popular Fingerprint Positioning Algorithms

Fingerprint-based k-nearest neighbors (KNN) indoor positioning was proposed by Bahl and Padmanabhan in 2000. The algorithm finds K RPs with the smallest Euclidean distance from TP in the fingerprint space and uses their mean coordinate as the TP’s estimation coordinate [11]. The WKNN algorithm assigns weighting coefficients to the position coordinates of different RPs based on KNN, and the weight of each RP is usually set as the reciprocal of the Euclidian distance between RP’s and TP’s fingerprints. WKNN improves the positioning accuracy, and the implementation is simple [12]. However, room remains for improvement, which has inspired much research. Proposed by Liu, M-WKNN uses a weighting coefficient algorithm based on the signal propagation model which revealed the nonlinear relationship between RSS and physical distance [13]. Roos proposed the GK algorithm, which realizes the estimated coordinates by calculating the mean value of K coordinates of RPs with the maximum likelihood probability [14]. The algorithm can achieve superb positioning accuracy, at the cost of much calculation.

More accurate estimation algorithms can improve positioning accuracy, but often have higher time complexity. LS-SVM indoor positioning transforms the positioning problem to one of multi-class classification [15,16]. Its regularization and kernel parameters (c, g) are determined through parallel grid searching. LS-SVM improves positioning accuracy but needs much training time. K-means clustering is more suitable for pre-classification of a fingerprint database. When used for estimation, it is the same as SVM, which increases complexity without a significant improvement in accuracy. Due to the large amount of data required, the sensitivity to fluctuating data, and high time complexity, deep neural networks are rarely used for indoor positioning.

Considering accuracy and speed comprehensively, KNN has achieved brilliant results in comparative experiments [19] and positioning competitions [20]. Therefore, the improvement of many localization algorithms is based on KNN algorithm. Both dynamic selection of the appropriate K value and assign weight to K nearest RPs may help to improve the efficiency of the algorithm.

## 3. Details of Proposed Algorithm

**Figure 1:**
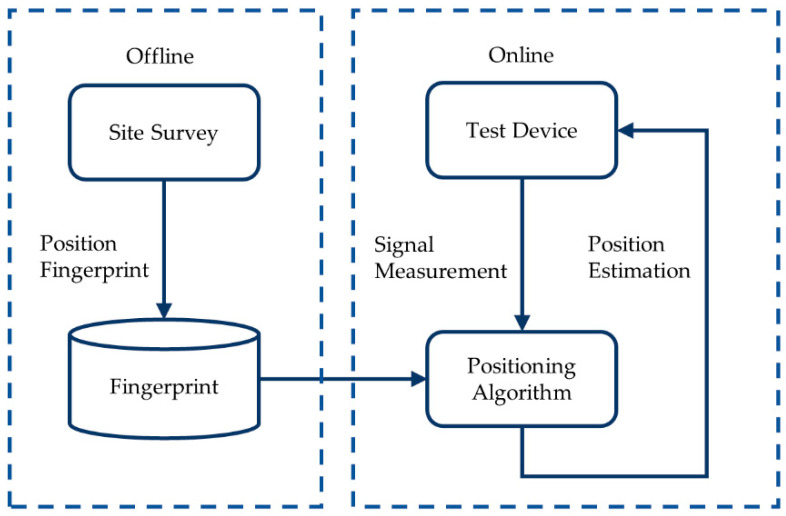
Flowchart of fingerprint positioning algorithm.

The Wi-Fi fingerprint positioning algorithm uses the RSS similarity between TP and RPs to obtain nearby RPs and then estimates the TP’s position based on their coordinates. As shown in Figure 1, the algorithm has two phases: offline training and online positioning.

The offline training phase, or site survey, constructs a fingerprint database consisting of preset RP coordinates and RSS, which is time- and labor-consuming. The Wi-Fi signal detection device collects the RSS corresponding to each Wi-Fi signal AP and generates a fingerprint database. In addition, due to the characteristics of signal propagation, RSS is easily affected by environmental changes, which causes unstable values. Hence, the RSS of each RP must be measured multiple times. The online positioning phase matches the fingerprints of TPs and RPs in the database and estimates the TPs’ positions according to the matching results. The higher the fingerprint similarity between TP and RP, the closer they are.

### 3.1. Fingerprint and Database

The combination of collected RSS is called a fingerprint, which could be classified as that of a TP or RP. A TP’s fingerprint is measured in the online phase and used to estimate the TP’s position, while an RPs’ fingerprint stored in the database plays the role of a predefined criterion.

Suppose $RSS_{\left(i,j\right)}$ represents the RSS from $AP_{i}$ received at $RP_{j}$, and the numbers of APs and RPs are n and m, respectively. Assume that the fingerprint acquired during the online phase is $FP_{TP}=\left[RSS_{\left(1,TP\right)},\;RSS_{\left(2,TP\right)},\ldots ,RSS_{\left(n,TP\right)}\right]$, and RSS_(i,TP)_ is the RSS received from $AP_{i}$.

To reduce the buffering influence, fingerprint collected at each RP are preprocessed before being saved. The fingerprint at the $RP_{j}$ is $FP_{j}=\left[RSS_{\left(1,j\right)},RSS_{\left(2,j\right)},\ldots ,RSS_{\left(n,j\right)}\right]$.

The fingerprint matrix FP is composed of fingerprints of each RP,

(1) $$FP=\left(\begin{array}{c} RSS_{\left(1,1\right)},RSS_{\left(2,1\right)},\ldots ,RSS_{\left(n,1\right)}\\ \begin{array}{c} RSS_{\left(1,2\right)},RSS_{\left(2,2\right)},\ldots ,RSS_{\left(n,2\right)}\\ \cdots \\ RSS_{\left(1,m\right)},RSS_{\left(2,m\right)},\ldots ,RSS_{\left(n,m\right)} \end{array} \end{array}\right).$$

As $RP_{j}$ has coordinates $\left(x_{j},y_{j}\right)$, the dataset includes the fingerprint and coordinates of each RP is named FPDB:

(2) $$FPDB=\left(\begin{array}{c} RSS_{\left(1,1\right)},RSS_{\left(2,1\right)},\ldots ,RSS_{\left(n,1\right)},x_{1},y_{1}\\ \begin{array}{c} RSS_{\left(1,2\right)},RSS_{\left(2,2\right)},\ldots ,RSS_{\left(n,2\right)},x_{2},y_{2}\\ \cdots \\ RSS_{\left(1,m\right)},RSS_{\left(2,m\right)},\ldots ,RSS_{\left(n,m\right)},x_{m},y_{m} \end{array} \end{array}\right).$$

### 3.2. RSS Fluctuation in Raw Fingerprint

**Figure 2:**
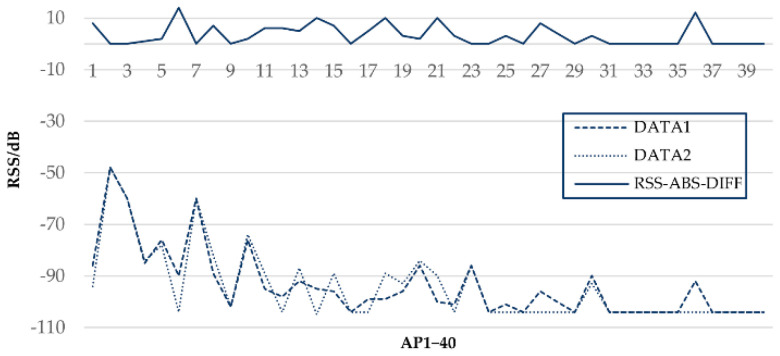
RSS difference of part AP (AP1−AP40) at the same position.

Similarity matrices commonly compare the RSS difference of the fingerprint between TP and RPs, whether large or small. However, in the actual environment, RSSs acquired from the same AP at the same position are different due to factors such as acquisition time, acquisition equipment, and environmental changes. For example, in Figure 2, DATA1 represents the RSS received at a position from AP1 to AP40, and DATA2 represents the RSS received at the same spot shortly after DATA1 under the same conditions. The absolute RSS difference between DATA1 and DATA2 is denoted as RSS-ABS-DIFF and is drawn at the top.

It can be inferred from Figure 2 that even under the same conditions, RSS acquired at intervals would have a maximum fluctuation of about 10 dB. The RSS difference of fingerprints always contains such fluctuations. Most algorithms do not account for their impact when calculating fingerprint similarity. Simply ignoring such fluctuations undoubtedly undermined the fingerprint similarity, which further affects positioning accuracy.

### 3.3. Data Preprocessing

The complexity of Wi-Fi signal propagation leads to fluctuations of RSS, i.e., the measured RSS at a given position fluctuates continuously. To build a robust fingerprint database, the raw RSS must be preprocessed to eliminate abnormal data and coarse errors.

Assume that $RSS_{\left(i,j\right)}$, which is the RSS of $AP_{i}$ at $RP_{j}$, is measured p times, and measurements collection is $S=\left\{RSS_{\left(i,j\right)}^{\left(1\right)},RSS_{\left(i,j\right)}^{\left(2\right)},\cdots ,RSS_{\left(i,j\right)}^{\left(p\right)}\right\}$.

We define the residual $r_{q}$ to indicate the degree of deviation of $RSS_{\left(i,j\right)}^{\left(q\right)}$ from the mean $\bar{RSS_{\left(i,j\right)}}$,

(3) $$r_{q}=RSS_{\left(i,j\right)}^{\left(q\right)}-\bar{RSS_{\left(i,j\right)}},q\in [1,p],$$

where $\bar{RSS_{\left(i,j\right)}}=\frac{1}{p}\overset{p}{\underset{q=1}{\sum }}RSS_{\left(i,j\right)}^{\left(q\right)}$. We calculate the root mean square error of the residual,

(4) $$\sigma =\sqrt{\left(\;\frac{1}{p}\sum_{i=q}^{p}\;r_{q}{}^{2}\right)},$$

Pauta criterion is suitable for eliminating the gross errors on large number of data samples that are approximately the normal distribution. In fact, the RSS measurement values have a slightly left-skewed normal distribution, which generally could be regarded as approximately normal [36]. As the amount of a $RSS_{\left(i,j\right)}$ measurement in our experiment is large (up to 300), we take Pauta criterion to eliminate gross error.

Therefore, according to the Pauta criterion, in the normal distribution, σ is the standard deviation, μ is the mean, and the probability that the value in (μ−3σ,μ+3σ) is 99.73%. If a residual satisfies $\left|r_{q}\right|>3\sigma$, then the corresponding $RSS_{\left(i,j\right)}^{\left(q\right)}$ is removed from S as a coarse error. The S without coarse error is named S’. To improve the robustness of the fingerprint, the average of preprocessed S’, which eliminates abnormal data and coarse errors, is taken as the final RSS.

More detailed fluctuation processing is required in the fingerprint matching stage, as we discuss next.

### 3.4. RSS Propagation and Base Q

To smooth the RSS fluctuation difference of fingerprint, in this part, base Q is introduced to transform RSS to Q-based RSS, after the relationship between the received signal strength (RSS) and physical distance in signal propagation is analyzed.

The Wi-Fi signal propagation model [37] is

(5) $$R_{\left(d_{i}\right)}=R_{\left(d_{0}\right)}-\eta 10\log_{10}\left(\frac{d_{i}}{d_{0}}\right)\pm X,$$

where $R_{\left(d\right)}$ is RSS at a point where is d meter away from the AP, η is the path loss exponent, and X is the RSS fluctuation caused by acquisition equipment, time, and environmental changes at the same place. $d_{0}$, $R_{\left(d_{0}\right)}$, and η are preset modeling parameters. $R_{\left(d_{i}\right)}$ is the measured RSS of $AP_{i}$ at a point where it is $d_{i}$ meter away from $AP_{i}$. According to the signal propagation model, as long as $R_{\left(d_{i}\right)}$ is known, the unknown distance, $d_{i}$, between the point and $AP_{i}$ could be calculated as

(6) $$d_{i}=d_{0}10^{\left(\frac{R_{\left(d_{0}\right)}-R_{\left(d_{i}\right)}\pm X}{10\eta }\right)}.$$

Assume RP and TP are two points that are $d_{i}^{RP}$ and $d_{i}^{TP}$ away from $AP_{i}$, respectively, then the physical distance Δd between RP and TP could be expressed as

(7) $$\begin{array}{cc} \Delta d|= & d_{i}^{RP}-d_{i}^{TP}\\ |= & d_{0}10^{\left(\frac{R_{\left(d_{0}\right)}-R_{\left({d_{i}}^{RP}\right)}\pm X^{RP}}{10\eta }\right)}-d_{0}10^{\left(\frac{R_{\left(d_{0}\right)}-R_{\left({d_{i}}^{TP}\right)}\pm X^{TP}}{10\eta }\right)}\\ |= & d_{0}10^{\frac{1}{10\eta }\left(R_{\left(d_{0}\right)}-R_{\left({d_{i}}^{RP}\right)}\pm X^{RP}\right)}-d_{0}10^{\frac{1}{10\eta }(R_{\left(d_{0}\right)}-R_{\left({d_{i}}^{TP}\right)}\pm X^{TP})}. \end{array}$$

Simplify ‘$-R_{\left(d_{i}^{RP}\right)}\pm X^{RP}$’ to $RSS^{RP}$.

Simplify ‘$-R_{\left(d_{i}^{TP}\right)}\pm X^{TP}$’ to $RSS^{TP}$.

Simplify $10^{\left(\frac{1}{10\eta }\right)}$ to *Q*, and Equation (7) is converted to

(8) $$\frac{\Delta d}{d_{0}}=Q^{R_{\left(d_{0}\right)}+RSS^{RP}}-Q^{R_{\left(d_{0}\right)}+RSS^{TP}}.$$

Simplify $Q^{R_{\left(d_{0}\right)}}$ to a.

Equation (8) is converted to

(9) $$\frac{\Delta d}{a\cdot d_{0}}=Q^{RSS^{RP}}-Q^{RSS^{TP}}.\;$$

Due to noise and missing signals, many believe that Euclidian distance is not the best measure of similarity [27]. In fact, fingerprints are not mathematic vectors defined in Euclidean space but a collection of RSS measurements, so Euclidean distance is not desirable for similarity measurement of collection with noise and missing elements. Research indicates that for a Wi-Fi fingerprint system, Euclidean distance for NN, and Manhattan distance for KNN or WKNN gave the least mean distance error [38]. Accordingly, we chose Manhattan distance as our fingerprint similarity measurement. However, the relationship between physical distance and signal differences is not directly proportional, and simple signal subtraction, containing the fluctuation, cannot directly reflect the difference in physical distance. Therefore, in order to smoothen the impact of severe fluctuations, we use Q-based RSS subtraction, instead of direct RSS subtraction, to compare similarity.

### 3.5. AP Selection Algorithm

In an ideal indoor environment, positioning results should be improved by using more APs [32]. However, in an actual indoor environment, the RSS of each AP is affected by obstacles and multipath effects. Using RSS from all detected APs without screening decreases positioning accuracy. Therefore, an algorithm is applied to select those APs that contribute more to positioning. Those RSSs from these APs increased positioning accuracy and decreased computational cost. The criterion for AP selection uses two indicators, $M\left(AP_{i}\right)$ and $P\left(AP_{i}\right)$, representing the maximum value of RSS and appearance ratio of $AP_{i}$, respectively.

Because the positioning accuracy using the average value of RSS as a feature is not as good as the maximum RSS in a steady-state environment [35]. The first indicator is

(10) $$M\left(AP_{i}\right)=\frac{\max_{AP_{i}}+U}{U},$$

where $\max_{AP_{i}}$ is the maximum value of RSS from $AP_{i}$, and U is the preset absolute value of the RSS of undetected APs. A larger $M\left(AP_{i}\right)$ indicates a more reliable $AP_{i}$.

The second indicator, $P\left(AP_{i}\right)$, is the appearance ratio of the signal of $AP_{i}$ in one complete fingerprint collection,

(11) $$P\left(AP_{i}\right)=\frac{S}{S-P_{AP_{i}}}$$

In one collection, S is the number of fingerprints in the dataset, and $P_{AP_{i}}$ is the times of $AP_{i}$’s appearance. $P\left(AP_{i}\right)$ increases with $P_{AP_{i}}$. When $P_{AP_{i}}=S$, $AP_{i}$ will be selected directly.

The criterion for AP selection reflects the reliability of the $AP_{i}$ and is defined as

(12) $$R\left(AP_{i}\right)=M\left(AP_{i}\right)*P\left(AP_{i}\right).$$

The APs are sorted in descending order of $R\left(AP_{i}\right)$, and the first L APs are selected for positioning.

### 3.6. Adaptive K Algorithm

In WKNN, the value of K plays an important role in positioning accuracy. A large K means more neighbor RPs including irrelevant RPs which lead to low positioning accuracy, and a small K implies the degradation of KNN. Our adaptive K is dynamically determined based on the fingerprints’ Manhattan distance collection, S, between RPs and TP. There are two filter steps in determining K.

First, a threshold KTh is set to filter each element in S, where S is a distance collection with m elements (m is the number of RPs). If the value is greater than KTh, then the element is removed from S. KTh should obviously be adaptive because every TP has a unique S. To reduce the error caused by a constant threshold, we use $KTh=2*S_{min}$, where $S_{min}$ is the minimum value in S. The filtered distance collection,

(13) $$S^{\prime }=\left\{S_{1},S_{2},\ldots ,S_{f}\right\}=\{S_{i}|S_{i}\leq KTh,1\leq i\leq m\},$$

contains f (f ≤m) elements in ascending order, corresponding to RPs.

Second, we define $G_{i}$ as $G_{i}=\left|S_{i}-S_{1}\right|$ ($S_{1}$ is the minimum), where i takes values from 2 to f. We calculate the mean of these differences,

(14) $$E\left(G\right)=\frac{\sum_{i=2}^{f}G_{i}}{f-1},$$

If $G_{i}$ is greater than E(G), then $S_{i}$ is removed from S′. After these comparisons are made, the number of elements remaining in *S*′ is the adaptive K, $K=Count\left(S^{\prime }\right)$.

### 3.7. Q-WKNN

**Figure 3:**
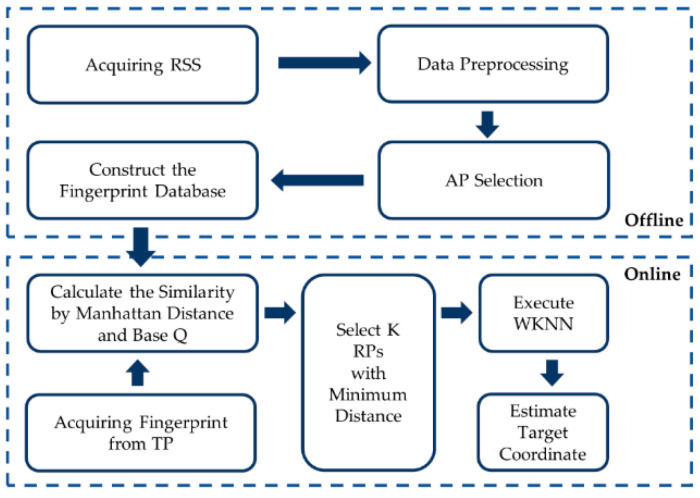
Flowchart of proposed algorithm.

According to the above analysis, to smooth the signal fluctuation and improve positioning accuracy, Q-based RSS Manhattan distance is adopted in the fingerprint similarity calculation between the RP and TPs after data preprocessing and AP selection. A TP’s position could be estimated by WKNN with adaptive K, as shown in the flowchart in Figure 3.

In summary, Q-based distance collection $D_{J}=\left\{d_{\left(1,j\right)},d_{\left(2,j\right)},\cdots ,d_{\left(L,j\right)}\right\}$ (where 1 to L indicate L reliable APs), consisting of the selected APs’ RSS difference between $RP_{j}$ and TP is adopted as a similarity metric.

As mentioned in Section 3.4, the base Q is introduced to the Manhattan distance to smooth the RSS fluctuation. Before using Manhattan distance to calculate similarity, according to Equation (9), Q is taken as the base number, and RSS is the index. Q-based RSSs are subtracted to map the physical distance difference,

(15) $$d_{\left(i,j\right)}=\left|Q^{RSS_{\left(i,j\right)}}-Q^{RSS_{\left(i,TP\right)}}\right|,i\in \left(1,L\right).$$

Accordingly, the fingerprints’ Manhattan distance between $RP_{j}$ and TP is

(16) $$S_{j}=\sum_{i=1}^{L}d_{\left(i,j\right)}.$$

After picking up the adaptive K-nearest neighbor RPs, the reciprocals of the Q-based Manhattan distances are taken as the weights collection,

(17) $$W=\left\{w_{1},w_{2},\ldots ,w_{k}\right\}=\left\{\frac{1}{S_{1}},\frac{1}{S_{2}},\ldots ,\frac{1}{S_{k}}\right\}$$

If the subscripts of the selected K RPs are [1,  K], the coordinates of $RP_{j}$ (1 ≤ *j* ≤ *K*) are $\left(X_{j},Y_{j}\right)$, and its corresponding weight is $w_{j}$, the TP’s coordination (X,Y) can be estimated as

(18) $$X=\frac{\sum_{j=1}^{K}(X_{j}w_{j})}{\sum_{j=1}^{K}w_{j}}\;Y=\frac{\sum_{j=1}^{K}(Y_{j}w_{j})}{\sum_{j=1}^{K}w_{j}}.$$

## 4. Experiment and Discussion

### 4.1. Experiment Environment

To test the positioning performance of Q-WKNN, we adopted two real-world datasets in different environments for experiment. Meanwhile, in order to check Q’s role, we also generated simulated data. The Zenodo dataset was provided by Mendoza-Silva, Richter, and Torres-Sospedra. We collected the Park dataset in the underground garage of North China Electric Power University. Zenodo is a massive, open, long-term database that is updated monthly with data acquired from the third- and fifth-floor bookshelf areas of a library. Since the two floors are identical in structure, we only used the third-floor data for experiment. The dataset contains 25 months of measurement data with 48 RPs (24 RPs per floor) RSS from a total of 620 APs (including APs whose signal were not detected in some month label). Each AP is uniquely identified by its media access control (MAC) address and service set identifier (SSID). The RSSs were acquired six times at each point to avoid error due to chance. To clearly name 25 months in a dataset, Zenodo uses numbers 1–25 to label these months.

There are three reasons for choosing the Zenodo dataset: (1) it provides up to 25 months of signal data, which enables researchers to fully test the reliability and stability of a positioning algorithm; (2) it includes several scenario simulations and many survey and test spots, such as a user’s stopping and walking state; (3) the dataset has been widely used for accuracy testing of positioning algorithms, making it convenient for comparison.

The Park dataset was used to compare the applicability of the Q-WKNN algorithm in different environments and determine the scope of the base Q. It uses a measurement method like Zenodo, but contains more actual environmental information, like the coordinates of every APs. The Park dataset uses a smaller number of fixed APs, and the coverage area of TPs is wider. Due to the frequent entry and exit of vehicles, the environmental changes in the dataset are more complicated and noisier.

**Figure 4:**
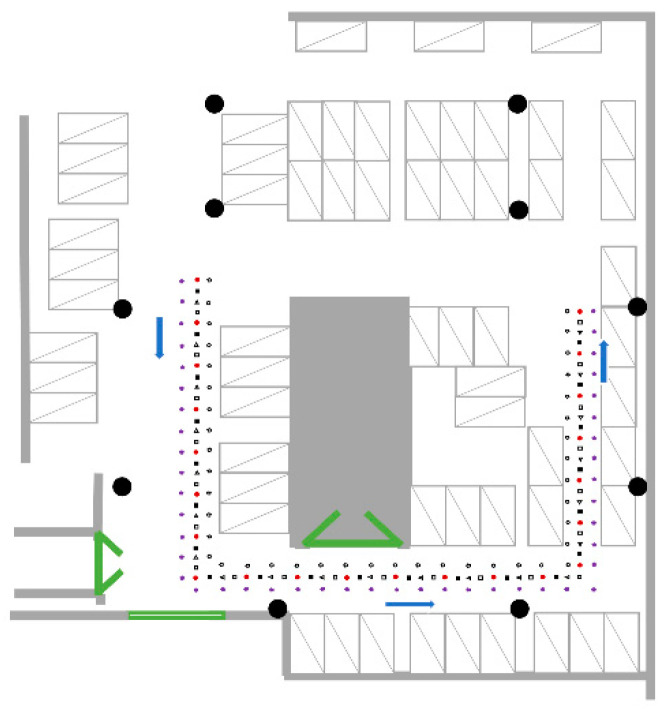
Schematic diagram of Park environment.

As show in Figure 4, ten wireless router devices (taken as APs in this paper), denoted by black circle, were used for Park dataset collection. Each device has both 2.4G and 5G frequency band signals, forming a total of 20 APs. RPs (denoted as 41 white pentagons and 45 purple pentagons) and TPs (denoted as 22 red dots, 21 black rectangles, 21 white triangles, and 21 white rectangles) are distributed in the U-shaped corridor, and measurement was done 10 times at each point. The U-shaped corridor surrounds the entrance of the underground garage, and the wall in the middle (denoted by gray rectangular) forms a rectangular area where Wi-Fi signals pass with difficulty. The test set was not derived from the training set but from separately selected TPs for a more credible test effect.

Table 1 compares the Zenodo and Park datasets.

### 4.2. Results and Comparison

In this paper, proposed algorithm Q-WKNN is the improvement of WKNN. Hence, WKNN, M-WKNN, and the common positioning algorithms GK and LS-SVM were compared with Q-WKNN to verify its positioning accuracy and real-time performance. Simulation on the Zenodo dataset proceeded as follows. For each month, positioning was performed using one training set as an RP dataset and all test sets as TPs to imitate different users. We calculated the positioning error and plotted the cumulative distribution function (CDF) for all test sets. Positioning accuracy was evaluated in terms of 75th percentiles of positioning error CDF. We used this instead of the mean error because the latter tends to be small, from which researchers may make overly optimistic judgments on the positioning accuracy of an algorithm. This standard is also used in a competition for the Indoor Positioning and Indoor Navigation (IPIN) conference.

The experiment was also carried out on the Park dataset, following the same procedures.

According to the principle of the proposed algorithm, its positioning accuracy is related to the base Q, the reliable AP number L, and adaptive K. Therefore, before comparing Q-WKNN with other algorithms, the effects of the hyperparameters Q, K, and L are subject to experiment, respectively.

#### 4.2.1. Impact of base Q

The effect of Q was inferred on the simulated data and tested on real data for verification. According to Equation (8), Q is calculated as $Q=10^{\left(\frac{1}{10\eta }\right)}$, where η is the path loss factor.

The algorithm used in the following experiment is KNN, the K value is 3, and the similarity measure is Manhattan distance. The same data preprocessing steps, mentioned in Section 3.3, are used for each set of simulated data and real data.

As Q is inferred from a signal propagation model, we first verify the smoothing effect of Q on simulated data. The source of signal fluctuation dominated by thermal noise is complex and changeable, so we superimpose Gaussian noise and use additive noise to simulate signal fluctuations caused by a real environment.

**Figure 5:**
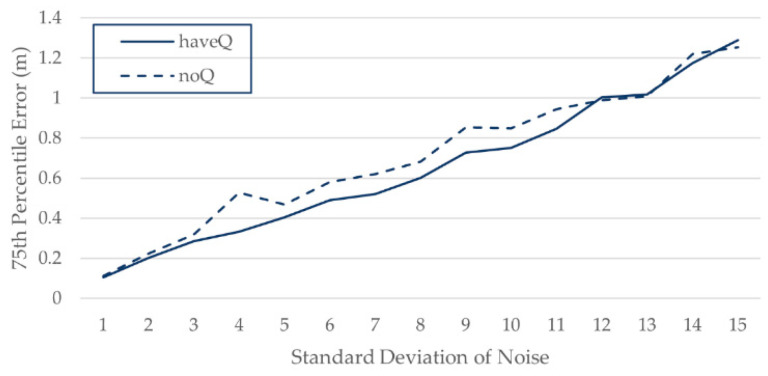
Positioning error of different noise standard deviations on simulated data.

The simulation area was 60 m long and 30 m wide, and 12 APs were randomly set. To imitate an actual situation as realistically as possible, some APs could not be detected at some spots. A signal strength less than −90 dB was considered undetectable. Fifteen sets of data were generated in this area, with noise standard deviations varied from 1 dB to 15 dB, and η was set to 4.5. As shown in Figure 5, when the noise standard deviation is 2–11 dB, the data processed by base Q can decrease the position error. Data with excessive noise (bigger than 12 dB) makes it difficult to extract effective features, and the effect of base Q is not obvious when the noise is too small (less than 2 dB).

**Figure 6:**
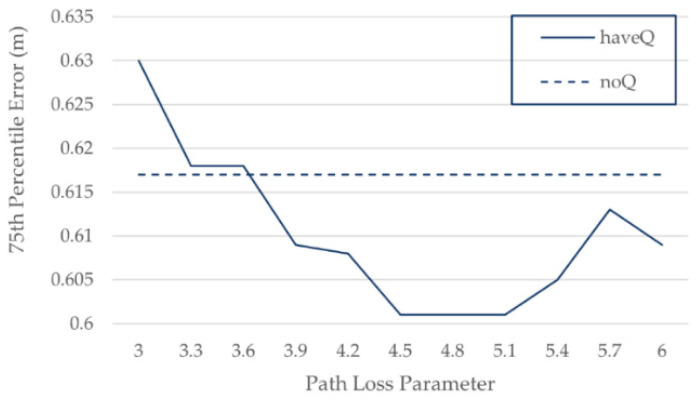
Positioning error of different noise standard deviations on simulated data.

Signals from different AP have different paths to TP, and the corresponding η is also different. In addition, the value of η is often unknown in the real world. In order to simulate the unknown state of η in the real world, we assumed that η was unknown and experimented with Q corresponding to different values of η in the range 3–6. It can be seen from Figure 6 that the improvement of positioning accuracy becomes insignificant or even decreases when η deviates greatly from the true value of 4.5, and the base Q has a certain smoothing effect when η is 3.9–5.4, which is around the true value of 4.5.

Based on the above speculation, experiments were conducted on the Zenodo and Park datasets. In fact, η is an empirical parameter, and its common value is 2–6 in an indoor environment. In an actual environment, it is difficult to accurately determine η for signals transmitted by each AP, and only its η distribution can be determined empirically.

**Figure 7:**
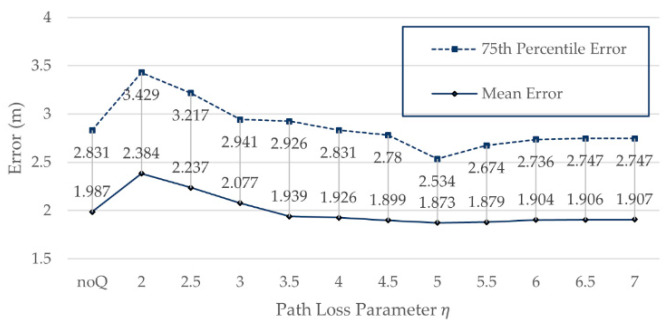
Positioning errors of different values of *η* on Zenodo.

We experimented with Q corresponding to different values of η in the range 2–7 on the Zenodo dataset. The positioning errors of different values of η on Zenodo are shown in Figure 7.

It can be seen from Figure 7 that when the value of η is 5, 75%, positioning error and mean error achieved their highest improvements, 2.534 m and 1.873 m, respectively, and after this point, there is no obviously improvement. Therefore, η = 5 is the best value in this environment. When η is near 5, compared with not using base Q processing, a certain accuracy improvement is obtained. The above result is consistent with our speculation from the simulated data that the positioning error can be decreased when the η used to calculate Q is near the true η.

We also used data from other month labels in Zenodo to conduct experiments. In months labeled 06, 08, 11, 14, and 15, Q failed and even brought more errors. It is speculated that Zenodo contains up to 620 APs, a large number of them are useless, and effective APs are constantly changing. In addition, in the months when Q fails, effective AP values less than −90 dB and more than −30 dB accounted for a higher proportion.

Unlike Zenodo, the Park dataset comes from 20 stable AP signals. More than 80% of the RSS values are in the stable signal range of −50 dB to −90 dB, and the data are approximately Gaussian. The main factor affecting positioning accuracy is signal fluctuations caused by vehicles entering and exiting. With the three-week data of the Park dataset, the value of *η* was increased from 3 to 7 at intervals of 0.5. When η = 7, the three sets of data all achieved the best positioning effect. Table 2 shows the results of the three-week data processing using the base Q. It can be seen in three weeks, compared to WKNN, the 75% positioning error of Q-WKNN achieved 0.103 m, 0.329 m, and 0.409 m drop, respectively, which are 4.41%, 13.98%, and 19.38% decreases accordingly.

The positioning accuracy in the third week improved significantly, and the improvement in the first week was small, because the training sets of the second and third weeks were measured on working days and the first week was measured on rest days. The frequent entry and exit of vehicles in the underground garage during working days can better reflect the smoothing effect of the base Q on the signal.

In summary, the base Q is suitable for smoothing data with the following characteristics: (1) the signal can always be detected, and its value is within a meaningful range; (2) the signal fluctuates sharply due to environmental changes, which better reflects the smoothing effect of the base Q.

Therefore, AP selection plays an important role in positioning accuracy. We performed an analysis to select an optimal number L for AP selection.

#### 4.2.2. Impact of Reliable AP Number L

**Figure 8:**
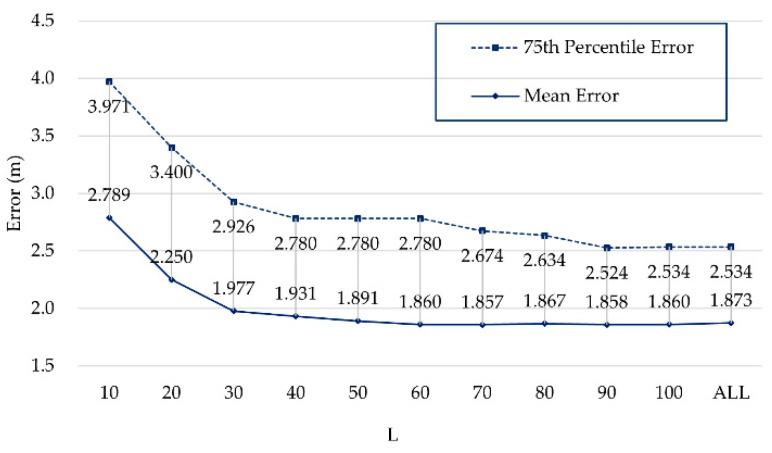
Positioning errors of different L on Zenodo.

The object of AP selection is to select APs that contribute more to positioning. It is important to find the optimal reliable number L of APs to improve positioning accuracy and reduce computation costs. We set L from 10 to 100 to see the impact on positioning and compared the results with the use of no AP selection algorithm, as shown in Figure 8. In this experiment, the total number of APs was 620, and “ALL” on the horizontal axis in Figure 8 means L = 620.

Initially, the positioning error gradually decreases as L increases, but it does not continue to decrease when L exceeds 90. It can be inferred from Figure 8 that setting an appropriately reliable AP number L can improve positioning accuracy. We take 90 as a reliable number L for AP selection.

#### 4.2.3. Impact of K in WKNN

Two adaptive filtering steps are used to obtain the dynamically changing K, and K undoubtedly affects the positioning accuracy. When K is 1, the algorithm degenerates to the NN algorithm.

**Figure 9:**
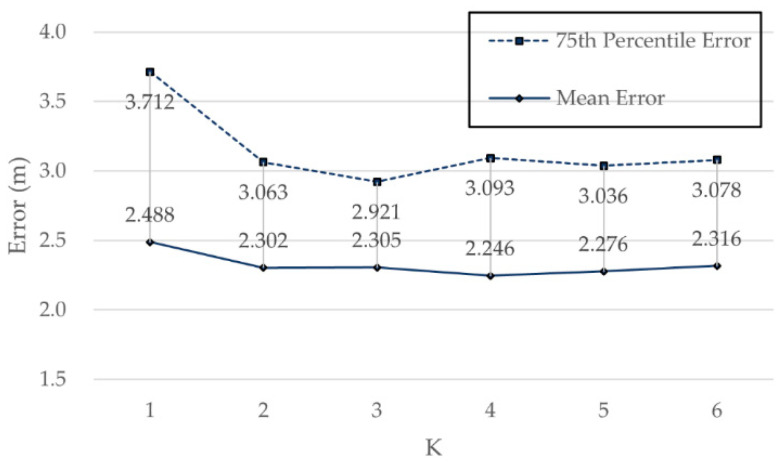
Positioning errors for different K based on WKNN on Zenodo.

Different K values were used to perform the positioning experiment based on normal WKNN, and the value with the highest positioning accuracy was found for subsequent comparison of algorithms. We set K from 1 to 6, with results as shown in Figure 9.

It can be seen from Figure 9 that the positioning accuracy of WKNN gradually increased with K, but it did not increase permanently. When K was 3, the error of the 75th percentile was 2.92 m. Therefore, subsequent experiment took K = 3 as the parameter of WKNN.

**Figure 10:**
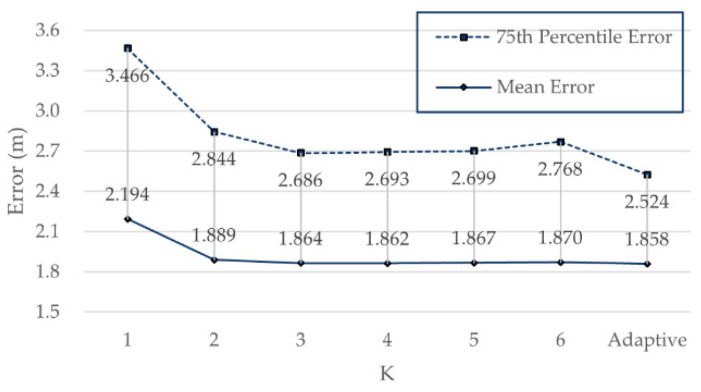
Positioning errors of different K and adaptive K based on Q-WKNN on Zenodo.

To verify the improvement of the positioning accuracy of the adaptive K algorithm, Q-WKNN based on different K and adaptive K was used for positioning. The results are shown in Figure 10.

As can be seen from Figure 10, the positioning accuracy of Q-WKNN rose with increasing K, but it did not rise permanently. When K was 4, the mean error with the biggest decrease was 1.862 m, which was still greater than the mean error of 1.858 m using the adaptive K. When K was 3, the 75th percentile error with biggest decrease was 2.686 m, which was still greater than that of 2.524 m using the adaptive K. Therefore, compared with a fixed K, Q-WKNN based on adaptive K brought an improvement in positioning accuracy.

#### 4.2.4. Positioning Accuracy Comparison of Algorithms

The proposed Q-WKNN algorithm was implemented with the commonly used WKNN, M-WKNN, GK, and LS-SVM on the Zenodo dataset. According to the above results, the hyperparameters of the algorithm were set as follows. For WKNN (M-WKNN), K = 3. For Q-WKNN, η = 7 and L = 90. For GK, K = 6 and σ = 4. For LS-SVM, (c,g) were automatically optimized.

The positioning error of the 75th percentile and the mean positioning error are shown in Table 3. Compared to WKNN, M-WKNN, GK, and LS-SVM, the positioning error of Q-WKNN decreased by 20.2%, 17.1%, 21.3%, and 21.8%, respectively.

Table 4 shows the cumulative error probability of different algorithms under a fixed accuracy limit, which is higher for Q-WKNN than for other algorithms under the same positioning accuracy limits.

The obtained results show that Q-WKNN has a certain improvement in positioning accuracy compared to WKNN, M-WKNN, GK, and LS-SVM.

#### 4.2.5. Time-Consumption Comparison of Algorithms

The proposed algorithm not only improves the accuracy but partly reduces the time consumption of positioning. AP selection in the offline phase reduces the number of AP used for fingerprint matching in the online phase. In addition, gross error elimination and fluctuation smoothing in advance in the offline stage also saves the time in the online stage.

The time needed in a single location is mainly contains the signal measurement and the delay of data transmission with the server. Otherwise, the time of fingerprint matching is very short, and it is difficult for users to perceive. From the view of the fingerprint matching algorithm alone, when it comes to large amount of positioning requests, the advantage of short time consumption could be better reflected. Therefore, we use the CPU time consumed by different positioning algorithms as a time-consumption criterion. Table 5 shows the time consumption of algorithms in Table 4 with different amount of test points. To avoid errors due to accidents, time-consumption experiments are carried out five times, and the average is taken. The time unit in Table 5 is second(s).

Referring to Table 5, when the total number of test points is small, the positioning time consumption is similar to that of algorithms other than GK, but the difference gradually increases with number increase. According to the result of 3900, the positioning time consumption of Q-WKNN, compared to WKNN, M-WKNN, GK, and LS-SVM, has decreased by 23.1%, 41.2%, 87.6%, and 47.4%, respectively.

In Table 5, it is clear that the positioning time consumption of Q-WKNN with different test point number is much less than that of other algorithms, and as the total number increases, the gap becomes more obvious. In summary, Q-WKNN is superior to the comparison algorithms in real-time performance.

## 5. Conclusions

We presented an RSS transform-based WKNN algorithm after smoothing signal fluctuations for Wi-Fi indoor positioning. After deducing the relationship between physical distance and RSS according to a signal propagation model, the base Q was introduced to smooth RSS fluctuation. As Manhattan distance valued every element’s contribution at the same degree, it is adopted as a similarity measurement to compare our Q based RSS transformations with direct RSS in aspect of the fluctuation’s smoothing. In addition, AP selection and an adaptive K algorithm were proposed to further improve the positioning accuracy and real-time performance of Q-WKNN. To verify the algorithm’s effectiveness and application range, experiments were carried out on two datasets with different characteristics. The results show that Q-WKNN has better positioning accuracy than common algorithms WKNN, M-WKNN, GK, and LS-SVM, and it consumes much less positioning time. In conclusion, the algorithm is suitable for areas where the AP is relatively fixed, and its superiority is better reflected when the signal fluctuates sharply due to environmental changes. While the proposed algorithm achieves several improvements, there is still room to increase positioning accuracy, such as to separately consider data on rest days and working days to reduce positioning errors.

## Figures and Tables

**Table 1:** Comparison of Zenodo and park datasets.

Attribute	Zenodo	Park
Distribution of RPs	Small area, zigzag route	Large area, U-shaped corridor
Major obstruction	Book rack	Concrete column, automobile
Dynamic change	Movement of people	In and out of vehicles
Missing values	Many	Few
Data distribution	Left-skewed normal	Approximately normal

**Table 2:** Q effect on three weeks of Park database.

Week	WKNN (m)	Q-WKNN (m)	Decrease (m)	Percentage of Decrease
1	2.339	2.236	0.103	4.41%
2	2.357	2.028	0.329	13.98%
3	2.108	1.700	0.409	19.38%

**Table 3:** Positioning errors of different algorithms on Zenodo.

Algorithm	Q-WKNN	WKNN	M-WKNN	GK	LS-SVM
Mean Error (m)	1.858	2.331	2.241	2.362	2.376
75th Percentile Error (m)	2.524	3.075	3.085	3.19	3.208

**Table 4:** Cumulative error probability of different algorithms under fixed accuracy limit on Zenodo.

Algorithm	1 m	1.5 m	2 m	2.5 m	3 m
Q-WKNN	26.28%	47.92%	59.42%	73.54%	82.21%
WKNN	15.36%	30.49%	46.41%	60.38%	73.26%
M-WKNN	15.31%	37.18%	47.18%	65.95%	73.69%
GK	19.18%	36.47%	49.01%	63.04%	72.02%
LS-SVM	16.15%	30.58%	46.05%	59.36%	70.8%

**Table 5:** Time consumption for algorithms based on different test sample sizes on Zenodo.

	Total Number of Test Points	260	780	1300	2860	3900
Algorithm						
WKNN		0.1	0.4	0.8	1.7	2.6
M-WKNN		0.1	0.5	1.1	2.3	3.4
Q-WKNN		0.1	0.3	0.6	1.3	2.0
GK		1.0	3.3	5.6	12.4	16.2
LS-SVM		0.2	0.5	1.2	2.6	3.8

## Data Availability

The experiment uses an internal data set and a public data set Zenodo. The data presented in this study are available on request from the corresponding author.

## References

[1] Xie Y., Wang Y., Nallanathan A., Wang L. (2016). An improved K-nearest-neighbor indoor localization method based on spearman distance. IEEE Signal Process. Lett..

[2] Sun D., Wei E., Ma Z., Wu C., Xu S. (2021). Optimized CNNs to Indoor Localization through BLE Sensors Using Improved PSO. Sensors.

[3] Ashraf I., Hur S., Park Y. (2019). Indoor positioning on disparate commercial smartphones using Wi-Fi access points coverage area. Sensors.

[4] Krapež P., Vidmar M., Munih M. (2021). Distance Measurements in UWB-Radio Localization Systems Corrected with a Feedforward Neural Network Model. Sensors.

[5] Liu M., Wang H., Yang Y., Zhang Y., Ma L., Wang N. (2018). Rfid 3-d indoor localization for tag and tag-free target based on interference. IEEE Trans. Instrum. Meas..

[6] Bai S., Luo Y., Wan Q. (2020). Transfer Learning for Wireless Fingerprinting Localization Based on Optimal Transport. Sensors.

[7] He S., Chan S.-H.G. (2015). Wi-Fi fingerprint-based indoor positioning: Recent advances and comparisons. IEEE Commun. Surv. Tutor..

[8] Torres-Sospedra J., Montoliu R., Martínez-Usó A., Avariento J.P., Arnau T.J., Benedito-Bordonau M., Huerta J. UJIIndoorLoc: A new multi-building and multi-floor database for WLAN fingerprint-based indoor localization problems. Proceedings of the 2014 International Conference on Indoor Positioning and Indoor Navigation (IPIN).

[9] Torres-Sospedra J., Jiménez A.R., Knauth S., Moreira A., Beer Y., Fetzer T., Ta V.-C., Montoliu R., Seco F., Mendoza-Silva G.M.J.S. (2017). The smartphone-based offline indoor location competition at IPIN 2016: Analysis and future work. Sensors.

[10] Mendoza-Silva G.M., Richter P., Torres-Sospedra J., Lohan E.S., Huerta J. (2018). Long-term WiFi fingerprinting dataset for research on robust indoor positioning. Data.

[11] Bahl P., Padmanabhan V.N. RADAR: An in-building RF-based user location and tracking system. Proceedings of the IEEE INFOCOM 2000. Conference on Computer Communications. Nineteenth Annual Joint Conference of the IEEE Computer and Communications Societies (Cat. No. 00CH37064).

[12] Gansemer S., Püschel S., Frackowiak R., Hakobyan S., Grossmann U. (2010). Improved RSSI-based Euclidean distance positioning algorithm for large and dynamic WLAN environments. Int. J. Comput..

[13] Liu W., Fu X., Deng Z., Xu L., Jiao J. Smallest enclosing circle-based fingerprint clustering and modified-WKNN matching algorithm for indoor positioning. Proceedings of the 2016 International Conference on Indoor Positioning and Indoor Navigation (IPIN), 2017.

[14] Roos T., Myllymäki P., Tirri H., Misikangas P., Sievänen J. (2002). A probabilistic approach to WLAN user location estimation. Int. J. Wirel. Inf. Netw..

[15] Pei L., Liu J., Guinness R., Chen Y., Kuusniemi H., Chen R. (2012). Using LS-SVM based motion recognition for smartphone indoor wireless positioning. Sensors.

[16] Wei Y., Wang D., Yan Z. Axial decoupled LS-SVMs for indoor positioning using RSS fingerprints. Proceedings of the 2015 34th Chinese Control Conference (CCC).

[17] Zhang C., Qin N., Xue Y., Yang L. (2020). Received signal strength-based indoor localization using hierarchical classification. Sensors.

[18] Ashraf I., Hur S., Park S., Park Y. (2020). DeepLocate: Smartphone based indoor localization with a deep neural network ensemble classifier. Sensors.

[19] Bozkurt S., Elibol G., Gunal S., Yayan U. A comparative study on machine learning algorithms for indoor positioning. Proceedings of the 2015 International Symposium on Innovations in Intelligent SysTems and Applications (INISTA).

[20] Lymberopoulos D., Liu J., Yang X., Choudhury R.R., Handziski V., Sen S. A realistic evaluation and comparison of indoor location technologies: Experiences and lessons learned. Proceedings of the 14th International Conference on Information Processing in Sensor Networks.

[21] Tse D., Viswanath P. (2006). Fundamentals of Wireless Communication.

[22] Youssef M., Agrawala A. Handling samples correlation in the horus system. Proceedings of the IEEE Infocom 2004—The Conference on Computer Communications—Twenty Third Annual Joint Conference of the IEEE Computer and Communications Societies.

[23] Lin K., Chen M., Deng J., Hassan M.M., Fortino G. (2016). Enhanced fingerprinting and trajectory prediction for IoT localization in smart buildings. IEEE Trans. Autom. Sci. Eng..

[24] Wu C., Xu J., Yang Z., Lane N.D., Yin Z. (2017). Gain without pain: Accurate WiFi-based localization using fingerprint spatial gradient. Proc. ACM Interact. Mob. Wearable Ubiquitous Technol..

[25] Fang S.-H., Lin T.-N., Lee K.-C. (2008). A novel algorithm for multipath fingerprinting in indoor WLAN environments. IEEE Trans. Wirel. Commun..

[26] Wang B., Zhou S., Liu W., Mo Y. (2014). Indoor localization based on curve fitting and location search using received signal strength. IEEE Trans. Ind. Electron..

[27] Torres-Sospedra J., Montoliu R., Trilles S., Belmonte Ó., Huerta J. (2015). Comprehensive analysis of distance and similarity measures for Wi-Fi fingerprinting indoor positioning systems. Expert Syst. Appl..

[28] Peng X., Chen R., Yu K., Ye F., Xue W. (2020). An Improved Weighted K-Nearest Neighbor Algorithm for Indoor Localization. Electronics.

[29] Li Z., Zhong X., Wei J., Shi H. The application of manhattan tangent distance in outdoor fingerprint localization. Proceedings of the 2018 IEEE Global Communications Conference (GLOBECOM).

[30] Du X., Yang K. (2016). A map-assisted WiFi AP placement algorithm enabling mobile device’s indoor positioning. IEEE Syst. J..

[31] Chen Y., Yang Q., Yin J., Chai X. (2006). Power-efficient access-point selection for indoor location estimation. IEEE Trans. Knowl..

[32] Deng Z., Ma L., Xu Y. Intelligent AP selection for indoor positioning in wireless local area network. Proceedings of the 2011 6th International ICST Conference on Communications and Networking in China (CHINACOM).

[33] Jhuang F.-M., Hung C.-F., Tuan C.-C., Wu Y.-C., Leu F.-Y. An AP selection with RSS standard deviation for indoor positioning in Wi-Fi. Proceedings of the 2015 9th International Conference on Innovative Mobile and Internet Services in Ubiquitous Computing.

[34] Miao H., Wang Z., Wang J., Zhang L., Liu Z.F. A novel access point selection strategy for indoor location with Wi-Fi. Proceedings of the 26th Chinese Control and Decision Conference (2014 CCDC).

[35] Xue W., Qiu W., Hua X., Yu K. (2017). Improved Wi-Fi RSSI measurement for indoor localization. IEEE Sens. J..

[36] Kaemarungsi K., Krishnamurthy P. Modeling of indoor positioning systems based on location fingerprinting. Proceedings of the IEEE INFOCOM 2004–The Conference on Computer Communications–Twenty Third Annual Joint Conference of the IEEE Computer and Communications Societies.

[37] Luo R.C., Hsiao T.J. (2018). Dynamic wireless indoor localization incorporating with an autonomous mobile robot based on an adaptive signal model fingerprinting approach. IEEE Trans. Ind. Electron..

[38] Moghtadaiee V., Dempster A.G. Vector distance measure comparison in indoor location fingerprinting. Proceedings of the International Global Navigation Satellite Systems Society (IGNSS Symposium).

